# *In Vitro* Activities of MCB3681 and Eight Comparators against Clostridium difficile Isolates with Known Ribotypes and Diverse Geographical Spread

**DOI:** 10.1128/AAC.02077-16

**Published:** 2017-02-23

**Authors:** Jane Freeman, Sally Pilling, Jonathan Vernon, Mark H. Wilcox

**Affiliations:** a Microbiology, Leeds Teaching Hospitals Trust, University of Leeds, Leeds, United Kingdom; b Healthcare Associated Infections Research Group, Leeds Institute for Biomedical and Clinical Sciences, University of Leeds, Leeds, United Kingdom

**Keywords:** Clostridium difficile, MCB3681, antimicrobial susceptibility

## Abstract

Treatments for Clostridium difficile infection remain limited, despite the introduction of fidaxomicin, and development of new agents is necessary. We determined the *in vitro* susceptibilities of 199 prevalent or emerging Clostridium difficile PCR ribotypes to MCB3681, a novel investigational quinolonyl-oxazolidinone, and 8 comparators (metronidazole, vancomycin, fidaxomicin, moxifloxacin, ciprofloxacin, clindamycin, tigecycline, and linezolid). MCB3681 showed good activity against C. difficile with no evidence of MCB3681 resistance in isolates showing either moxifloxacin or linezolid resistance or both moxifloxacin and linezolid resistance.

## TEXT

Clostridium difficile infection (CDI) is a major burden on health care resources. CDI is thought to arise following the depletion of gut microflora by antimicrobial action, allowing the organism to proliferate and cause disease. Antimicrobial treatments for CDI are currently limited to metronidazole, vancomycin, and fidaxomicin. Metronidazole has more recently been associated with treatment failures, while promotion of glycopeptide resistance within the host microflora is a risk associated with vancomycin therapy (1). Symptomatic recurrence is common following treatment with these agents (2), requiring further episodes of antimicrobial therapy. Further treatment options are highly desirable to broaden the range of therapeutic choice and strengthen antimicrobial stewardship.

MCB3681 is a novel small molecule with structural elements of an oxazolidinone and a quinolone showing good activity against C. difficile, including isolates that were resistant to linezolid, ciprofloxacin, moxifloxacin, and clindamycin (3). It achieves high fecal concentrations after intravenous infusions and has shown activity against Gram-positive components of the gut microflora in a clinical phase 1 study (4). The development of an intravenous treatment agent achieving high fecal concentrations would circumvent issues of rapid gut transit or of impaired delivery of orally administered agents due to ileus, particularly in patients with severe or protracted/multiple recurrent diarrheal episodes.

We determined the *in vitro* activities of MCB3681 and 8 comparators (metronidazole, vancomycin, moxifloxacin, ciprofloxacin, clindamycin, tigecycline, linezolid, and fidaxomicin) against a panel of 200 Clostridium difficile isolates of known PCR ribotypes (RTs) from 21 European countries (selected from the *Clos*ER study, July 2011 to April 2013, by kind permission of Astellas Pharma Europe) (5).

*In vitro* susceptibility testing was performed using a Wilkins-Chalgren agar incorporation method, as previously described (5, 6). Briefly, C. difficile test isolates and control strains (C. difficile ATCC 750057, C. difficile E4 PCR ribotype 010, Bacteroides fragilis ATCC 25285, Enterococcus faecalis ATCC 29212, and Staphlyococcus aureus ATCC 29213) were cultured anaerobically at 37°C for 24 h in Schaedler anaerobic broth prior to dilution to a 0.5 McFarland standard equivalence in prereduced sterile saline solution and inoculation onto antibiotic-containing and control Wilkins-Chalgren agar plates. Inoculated plates were incubated anaerobically at 37°C for 48 h.

MCB3681 is a quinolonyl-oxazolidinone antibacterial which has previously demonstrated good activity against C. difficile (3). All the CDI treatment agents, including MCB3681, showed good activity against the isolates tested (Table 1). Fidaxomicin was the most active treatment agent (Kruskal-Wallis *P* = <0.0001; geometric mean [GM] MIC = 0.05 mg/liter), followed by MCB3681 (*P* = <0.0001; GM MIC = 0.12 mg/liter) and then metronidazole (*P* = <0.0001; GM MIC = 0.33 mg/liter), with no evidence of resistance to any of these compounds (Table 1). Vancomycin was the least active (*P* = <0.0001; GM MIC = 1.02 mg/liter), but resistance was very scarce (1.5%; breakpoint = >8 mg/liter). Reduced metronidazole susceptibility (MIC = 4 mg/liter) was observed in only 1% of isolates. GM metronidazole MICs were elevated in RT027 (0.96 mg/liter) and RT106 (0.74 mg/liter) versus the GM metronidazole MIC for all isolates tested (0.33 mg/liter), in agreement with previous data (4).

**TABLE 1:** Susceptibility of 199 C. difficile isolates to MCB3681 and 8 comparators^*a*^

Antimicrobial	Breakpoints (mg/liter) (reference no.)	% S	% I	% R	MIC_50_ (mg/liter)	MIC_90_ (mg/liter)	MIC range (mg/liter)	Geometric mean MIC (mg/liter)													
								RT001 (*n* = 15)	RT002 (*n* = 14)	RT005 (*n* = 16)	RT014 (*n* = 16)	RT015 (*n* = 15)	RT017 (*n* = 16)	RT018 (*n* = 14)	RT020 (*n* = 15)	RT027 (*n* = 16)	RT078 (*n* = 16)	RT106 (*n* = 14)	RT126 (*n* = 16)	RT356 (*n* = 16)	All isolates (*n* = 199)
MCB3681	S < 4; R > 4 (3)	100			0.125	0.25	0.008 to 0.5	0.07	0.11	0.14	0.11	0.14	0.15	0.12	0.1	0.16	0.11	0.11	0.12	0.08	0.12
FDX	S < 1; RS > 1 (5)	100			0.06	0.125	0.004 to 0.25	0.02	0.06	0.06	0.07	0.06	0.04	0.06	0.06	0.09	0.05	0.09	0.06	0.04	0.05
MTZ	S < 2; I = 4; R > 8 (5)	99	1		0.25	1	<0.125 to 4	0.42	0.19	0.29	0.28	0.25	0.26	0.41	0.25	0.96	0.26	0.74	0.32	0.27	0.33
VAN	S < 2; I = 4; R > 8 (5)	96	2.5	1.5	1	2	0.5 to 8	0.79	0.87	1.16	0.88	0.87	0.74	1.49	0.75	1.14	0.92	1.1	1	2.28	1.02
MXF	S < 2; I = 4; R > 8 (5)	50.5	1	48	2	32	1 to >64	16	1.82	2	3.36	1.91	12.88	6.9	2.59	21.67	2.38	7.61	8.35	29.34	5.87
CIP	S < 8; RS > 8 (3)			100	64	256	8 to >128	111.43	27.86	37.12	39.74	26.6	86.67	110.33	36.44	206.14	34.9	81.98	72.88	245.15	66.27
CLI	S < 2; I = 4; R > 8 (5)	5.5	29.5	54	16	128	1 to >64	61.11	12.13	9.28	10.37	7.29	64	8.83	11.31	19.87	12.34	10.77	38.05	12.88	16.17
TGC	S < 4; RS > 4 (5)	100			0.06	0.06	0.03 to 0.125	0.03	0.04	0.04	0.05	0.04	0.06	0.04	0.05	0.05	0.05	0.04	0.06	0.04	0.05
LZD	S < 4; R > 4 (8)	78.9		21.1	4	8	2 to >64	10.08	4.39	5.66	4.36	4.19	7.03	4.42	4.76	5.19	5.42	4.42	4.56	4.76	5.16

a FDX, fidaxomicin; MTZ, metronidazole, VAN, vancomycin; MXF, moxifloxacin; CIP, ciprofloxacin; CLI, clindamycin; TGC, tigecycline; LZD, linezolid; S, sensitive; I, intermediate; R, resistant; RS, reduced susceptibility.

All isolates were resistant to ciprofloxacin according to the defined breakpoints (Table 1), and 48% of isolates, including at least one isolate in each RT group tested, showed moxifloxacin resistance. Highly elevated MICs of both moxifloxacin (≥32 mg/liter) and ciprofloxacin (≥128 mg/liter) were prevalent for RT001, RT027, and RT356. Clindamycin MICs were highest for RT001, RT017, and RT126 (GM MICs = 61.11 mg/liter; 64 mg/liter, and 38.05 mg/liter, respectively), but there was evidence of clindamycin resistance in all RTs tested (Table 1). There was no evidence of tigecycline resistance (range = 0.03 to 0.125 mg/liter; GM MIC = 0.05 mg/liter), in agreement with previous data (4) (Table 1). The majority of isolates (78.9%) were sensitive to linezolid (table 1), with a GM MIC of 5.16 mg/liter. RT001 and RT017 showed the highest GM linezolid MICs (10.08 mg/liter and 7.03 mg/liter, respectively). This also is in agreement with previous observations (6). Three RT017 isolates and two RT027 isolates showed dual quinolone-oxazolidinone resistance phenotypes and MCB3681 MICs of 0.5 mg/liter. We have previously reported that these isolates showed high-level resistance to chloramphenicol (Table 2) (5, 7). Marín et al. reported linezolid, chloramphenicol, erythromycin, and clindamycin resistance associated with the presence of the multidrug resistance gene *cfr* in C. difficile RT017, RT078, and RT126 isolates (8).

**TABLE 2:**
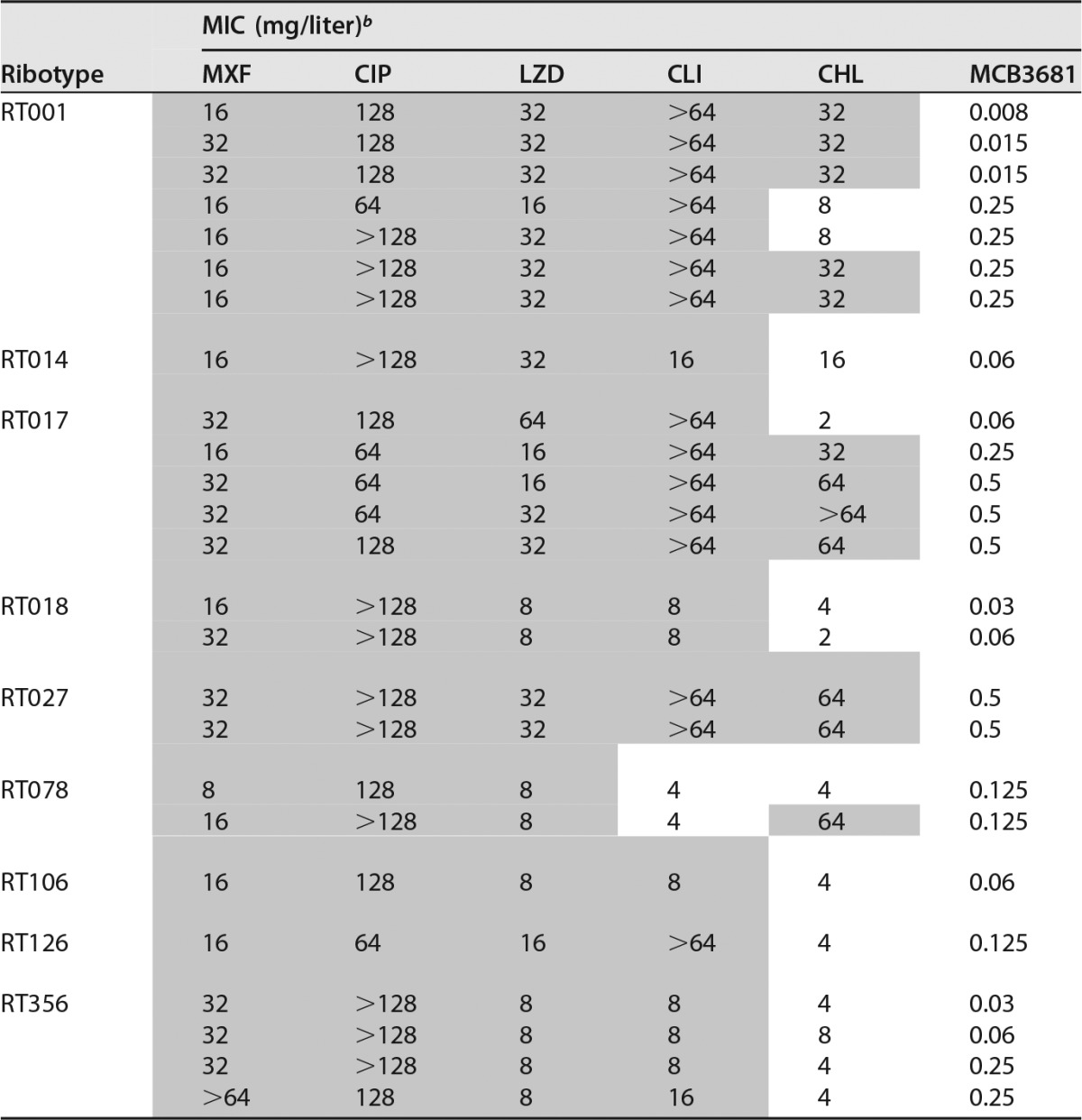
MCB3681 MICs in C. difficile isolates with dual quinolone-oxazolidinone resistance^*a*^

a Clindamycin and chloramphenicol MICs (5) are also shown. Highlighting indicates resistance.

^*b*^ MXF, moxifloxacin; CIP, ciprofloxacin; LZD, linezolid; CLI, clindamycin; CHL, chloramphenicol.

The MIC_50_ and MIC_90_ values reported here for MCB3681 are similar to those recently described for cadazolid, another quinolonyl-oxazolidinone molecule (9). A previous study investigating the susceptibility of C. difficile to cadazolid and comparators reported an association between resistance to either moxifloxacin or linezolid and moxifloxacin/linezolid doubly resistant mutants and 2- or 4-fold-higher cadazolid MICs in mono- or doubly resistant isolates, respectively (10). However, the highest MCB3681 MIC was 0.5 mg/liter, and we also found isolates with moxifloxacin, ciprofloxacin, linezolid, and chloramphenicol resistance that demonstrated very low MCB3681 MICs (0.008 mg/liter) (Table 2). We did not investigate the molecular basis of resistance in these isolates, but the results do not suggest a link between this phenotype and MCB3681 MICs. The results shown here, in conjunction with those previously reported (7, 8), would also seem to indicate that other modes of resistance to linezolid (23S rRNA alterations, ribosomal protein modifications) may be at play in combination with quinolone resistance mechanisms.

Rashid et al. reported that MICs of MCB3681 for C. difficile ranged from 0.008 to 0.5 mg/liter (3), which were values similar to our results (range, 0.008 to 0.5 mg/liter). However, in the present study, MIC_50_ and MIC_90_ values were 0.125 and 0.25 mg/liter, respectively, which were marginally higher than those reported previously but were within 2 doubling dilutions (0.03 and 0.06 mg/liter, respectively). This may be explained by methodology/agar or C. difficile strain distribution differences. The influence of testing media and components therein on MICs has previously been reported and may have been a factor in the differences observed (6, 11). We used a Wilkins-Chalgren agar incorporation method to determine MICs, since that method is superior to the use of CLSI-recommended brucella blood agar (BBA) in the detection of reduced susceptibility to metronidazole in C. difficile (6).

This report builds on the data previously reported by Rashid et al. by substantially expanding the diversity of ribotypes examined to include, in particular, RT027 and several RTs already noted for resistance to multiple antimicrobials: RT001, RT017, RT018, RT027, and RT356 (5, 7). There was no evidence of MCB3681 resistance among them. MCB3681 achieves fecal concentrations of 99 to 226 mg/kg of body weight after intravenous infusions, far in excess of the MIC ranges for C. difficile reported here. MCB3681 has been reported to be active against Gram-positive gut microflora bacteria but to be sparing of Gram-negative organisms in human volunteer studies with intravenous administration over 5 days. Further data are needed to assess the impact of MCB3681 on C. difficile and the gut microflora over a longer duration.

In summary, MCB3681 showed good activity against C. difficile isolates from emerging or prevalent European PCR ribotypes with no evidence of resistance. The presence of quinolone and/or linezolid resistance did not influence MCB3681 MICs.
